# Spectrum of heart diseases in children presenting to a paediatric cardiac echocardiography clinic in the Lake Zone of Tanzania: a 7 years overview

**DOI:** 10.1186/s12872-019-01292-4

**Published:** 2019-12-13

**Authors:** Antke Zuechner, Tumaini Mhada, Naizihijwa G. Majani, Godwin G. Sharau, William Mahalu, Matthias W. Freund

**Affiliations:** 1 Capacity Building Programme, CCBRT, Dar es Salaam, Tanzania; 2 Department of Paediatrics, Bugando Medical Centre, Mwanza, Tanzania; 3 Jakaya Kikwete Cardiac Institute, Dar es Salaam, Tanzania; 4 Department of Cardiac Surgery, Bugando Medical Centre, Mwanza, Tanzania; 5 Department of Paediatric Cardiology, University Hospital Oldenburg, Oldenburg, Germany

**Keywords:** Heart disease, Congenital, Acquired, Paediatric, Cardiac, Echocardiography, Tanzania

## Abstract

**Background:**

Congenital heart diseases (CHD) are among the most common congenital malformations. It is estimated that the incidence of CHD is constant worldwide, but data are rare for most African countries including Tanzania. Even less data are available on the prevalence of acquired heart diseases (AHD) in African children. Rheumatic heart disease (RHD) is the leading cause of AHD and is remaining a public health concern in Sub-Saharan Africa affecting especially the younger population. Both, CHD and AHD contribute substantially to morbidity and mortality during infancy and childhood.

**Methods:**

This hospital-based, retrospective review of the registry at the paediatric cardiac clinic of Bugando Medical Centre in the Lake Zone of Tanzania analysed the spectrum of heart diseases of paediatric patients during their first presentation by using simple descriptive statistics.

**Results:**

Between September 2009 and August 2016, a total of 3982 patients received cardiac evaluation including echocardiography studies. 1830 (46.0%) pathologic findings were described, out of these 1371 (74.9%) patients had CHD, whereas 459 (25.1%) presented with AHD.

53.9% of the patients with CHD were female and the most common associated syndrome was Down syndrome in 12.8% of patients. In 807 patients (58.9%) diagnosis of CHD was established within the first year of life. The majority of patients (60.1%) were in need of surgical or interventional therapy at time of diagnosis and 6.3% of patients were judged inoperable at the time of first presentation. Nearly 50% of cases with AHD were RHDs followed by dilated cardiomyopathy and pulmonary hypertension without underlying CHD.

**Conclusions:**

The spectrum of CHD and AHD from one centre in Tanzania is comparable to findings reported in other countries from the African continent. Echocardiography is a valuable diagnostic tool and the widespread use of it should be enhanced to diagnose heart diseases in a large number and reasonable time. Most patients present late and majority is in need of surgical or interventional treatment, which is still not readily available. Untreated heart diseases contribute substantially to morbidity and mortality during infancy and childhood. Adequate cardiac services should be established and strengthened.

## Background

Congenital heart diseases (CHD) include a variety of malformations of the heart and major vessels that are present at birth and that are among the most common congenital malformations [1, 2]. It is estimated that the incidence of CHD is constant worldwide [3, 4], though data are rare for most African countries [5, 6]. The estimate of 8 cases per 1000 live births is widely accepted with variations between regions and countries due to genetic and environmental differences [1, 7].

Little is known about the prevalence of CHD in Tanzania and only very few studies have been published [8, 9]. But given a current birth rate of 2.1 million babies per year in the United Republic of Tanzania [10] and considering an estimated incidence of 8 cases with CHD / 1000 live births would mean that up to 16.800 infants per year might be born with congenital heart disease.

Even less data are available on the prevalence of acquired heart diseases (AHD) in African children [11]. Rheumatic heart disease (RHD) is still the leading cause of AHD and is remaining a public health concern in Sub-Saharan Africa affecting especially the young population [11, 12].

Both form of heart diseases together contribute substantially to morbidity and mortality during infancy and childhood in low- and middle-income countries [6, 13]. We therefore report a 7 years overview describing the spectrum of heart diseases diagnosed in a paediatric cardiac echocardiography clinic in the Lake Zone of Tanzania.

## Methods

This hospital-based, retrospective study was conducted at the Bugando Medical Centre (BMC), Mwanza, Tanzania. BMC is one of four referral hospitals of mainland Tanzania serving for a population of approximately 14 million. The paediatric cardiac echocardiography clinic at BMC is performed twice per week, being the only clinic of its kind in the Lake Zone of Tanzania.

Patient data are recorded into a registration book and contain age, gender, type and severity of the heart disease as well as extracardiac malformations like Down syndrome (DS). Furthermore, a conclusion concerning the need for an intervention, e.g. surgery, is documented at the time of presentation. Children below 16 years of age who presented to the clinic for the first time and were diagnosed with an abnormal echocardiography study were included into the review. Cardiac consultation and echocardiography were performed by a paediatric cardiologist and by well-trained and supervised paediatricians (AZ, NM, TM). Echocardiography including two-dimensional-, colour-, pulse wave- and continuous wave-imaging was performed using two portable echocardiography machines, Sonosite M-Turbo and GE Loqic i. The leading lesion was recorded in cases of more than one cardiac lesion. The data were entered into Excel and then analysed by using simple descriptive statistics.

Quality control was ensured through visiting international cardiologists during cardiac missions and through intraoperative findings and diagnostic cardiac catheterizations done outside the country.

## Results

Between September 2009 and August 2016, a total of 3982 children underwent cardiac evaluation and echocardiographic examination at the paediatric cardiac clinic of BMC. In 1830 cases (46.0%) pathologic findings were described. Out of these, 1371 (74.9%) patients had CHD, and 459 (25.1%) presented with AHD.

### Congenital heart diseases

53.9% of the patients with CHD were female and 19.3% of all cardiac patients presented with associated syndromes or extracardiac malformations. The most common associated syndrome was DS in 12.8% of the patients. Leading cardiac lesion was ventricular septal defect (VSD) in 358 patients (26.1%) followed by patent ductus arteriosus (PDA) in 318 patients (23.2%), which included also 51 premature infants admitted to the neonatal department of BMC. Most common cyanotic heart disease diagnosed at our clinic was Tetralogy of Fallot (ToF) in 10.1% of cases. The distribution and frequencies of CHD are shown in Table 1.

**Table 1:** Distribution and relative frequencies of CHD

Diagnosis	Number (*N* = 1371)	%
Ventricular septal defect (VSD)	358	26.1
Patent ductus arteriosus (PDA) - PDA in premature infants - PDA in other children	318 51 267	23.2
Atrioventricular septal defect (AVSD) - AVSD in DS	144 98	10.5
Tetralogy of Fallot (ToF) - ToF with pulmonary atresia	138 16	10.1
Secundum Atrial septal defect	98	7.1
Pulmonary stenosis	58	4.2
Truncus arteriosus	50	3.8
Double outlet right ventricle	47	3.4
Complex cyanotic CHD	36	2.6
Tricuspidatresia	26	1.9
Transposition of great arteries (TGA) - TGA with large VSD	25 15	1.8
Others	73	5.3

Only 208 (15.2%) out of 1371 patients with CHD were diagnosed within the first month of life, by 6 months of life diagnosis was reached in 42% of patients and close to 60% of patients were at least diagnosed within the first year of life. Approximately 40% of the children with VSD as the leading CHD were diagnosed within the first 6 months of life, whereas children with ToF as the most common cyanotic CHD were usually diagnosed later in life, only one third being diagnosed within the first year of life. The overall age distribution at diagnosis and age distribution for VSD, PDA (excluding the PDA in premature infants) and ToF are presented in graph 1: age distribution for VSD, PDA, ToF and overall.

At time of diagnosis approximately 60% of patients were judged to be in need of surgical or interventional treatment. Another 96 patients (7.0%) were in need of diagnostic catheterization to assess eligibility for surgery. The largest sub-groups of patients for diagnostic catheterization were children diagnosed with AVSD (42 cases) beyond the first year of life followed by ToF with pulmonary atresia (16 cases), VSD (16 cases) and complex cyanotic heart diseases (8 cases).

In 41 patients (3.0%) signs of severe pulmonary arterial hypertension (PAH) were present at time of diagnosis. The largest group of patients with severe PAH were those with Truncus arteriosus communis diagnosed beyond the first year of life (18 cases), followed by VSD (7 cases) diagnosed beyond 15 months of life and AVSD (6 cases) diagnosed at the age between 5 and 11 years of life. The remaining patients who presented with severe PAH were diagnosed with AP-window, TAPVR (total anomalous pulmonary venous return), PDA, DORV (double outlet right ventricle) and TGA with large VSD.

The distribution of cases with respect to need of further diagnostic assessment or treatment is shown in Table 2.

**Table 2:** Need for further assessment or treatment

Condition	Number (*N* = 1371)	%
Mild CHD without need for surgery	392	28.6
Indication for surgery / intervention given	825	60.1
Diagnostic catheterization needed	96	7.0
Signs of severe pulmonary hypertension	41	3.0
Patient in critical clinical condition who died shortly after diagnosis	17	1.2

Seven patients (0,5%) were diagnosed with CHD while they presented to our hospital with acute infective endocarditis or endarteritis and intracardiac or intraarterial vegetations were demonstrated by echocardiography. All 7 patients were already pre-treated with different antibiotic regimes before referral to our centre and therefore, blood cultures taken on admission at BMC didn’t reveal the growth of any organism.

Nearly every fifth patient (19.3%) with congenital heart disease showed extracardiac malformations with DS being the leading associated syndrome. The distribution is depicted in Table 3.

**Table 3:** extracardiac malformations / syndromes

Condition	Number (*N* = 1371)	%
Down syndrome (DS)	176	12.8
Syndromic features (not classified)	35	2.6
Anorectal malformation	15	1.1
Multiple malformation	14	1.0
Rubella syndrome	12	0.8
Others^a^	13	0.9
Total number of associated malformations / syndromes	265	19.3

^a^Single cases of Omphalocele, Marfan Syndrome, Williams Syndrome, Turner Syndrome, connective tissue disease, Trisomy 18, missing sternum, ectopic Heart, and duodenal atresia

### Acquired heart diseases

Almost 50% of cases with AHD were RHD followed by dilated cardiomyopathy and pulmonary hypertension without an underlying structural heart disease. Table 4 is presenting the distribution and frequencies of AHD.

**Table 4:** Distribution and frequencies of AHD

Diagnosis	Number (*n* = 459 cases)	%	Additional information
Rheumatic Heart disease (RHD)	228	49.7	
Dilated Cardiomyopathy (DCM)	74	16.1	16 cases of HIV
Pulmonary Hypertension (PAH)	48	10.5	4 cases of Schistosomiasis
Pericardial effusion (PE)	33	7.2	10 cases of TB
Other cardiomyopathies	29	6.3	10 cases of malignancies
Persistent pulmonary hypertension of the newborn (PPHN)	27	5.9	
Endomyocardial Fibrosis (EMF)	20	4.3	

49.6% of the patients with RHD were female and the majority of patients diagnosed with RHD presented with isolated mitral valve regurgitation (69.3%), whereas 8.3% showed multivalvular disease followed by isolated aortic valve regurgitation (7.5%) and combined mitral valve regurgitation and mitral valve stenosis (6.6%)

At time of diagnosis most patients presented with advanced RHD and indication for surgery (valve repair or valve replacement) was given in 197 out of 228 cases (86.4%).

## Discussion

This is the first review on the spectrum of heart diseases in children in Tanzania and reports the data of a single centre. Out of 3982 patients who underwent cardiac evaluation and echocardiographic examination during the study period of 7 years 1830 (46%) presented with pathologic findings. Majority of patients (74.9%) were diagnosed with CHD. The overall distribution of heart diseases in congenital and acquired cases corresponds well with the findings of a comparable study in Cameroon, where out of 1666 cardiac patients, 73.8% presented with CHD and 25.8% with AHD [14].

Assessing the distribution of CHD in our review, VSD was the most common type, which is consistent with the results of two meta-analyses [1, 4] and a literature review [15] for the worldwide prevalence of this heart defect. Though most of the studies done in developing countries demonstrated even a higher prevalence of VSD ranging from 30 to 58% [14, 16–18] compared to our result of 26.1%.

As characteristic for countries with low resources and limited access to cardiac surgery Tetralogy of Fallot (ToF) is the leading cyanotic heart disease and the frequency is higher than documented in developed countries [14, 17–19]. Children with ToF do not present with heart failure, but with progressing signs of chronic cyanosis (e.g. finger clubbing) and frequent squatting. Many of them show a naturally balanced pathophysiology and survive without intervention whereas infants suffering from other cyanotic heart diseases might die early and even undiagnosed [17, 20]. This is also supported by our findings of diagnosis specific age distribution among children with VSD, PDA and ToF. Approximately 60% of children born with VSD or PDA were diagnosed within the first year of life, whereas the majority of children suffering from ToF seen in our centre (65.9%) was diagnosed beyond the first year of life.

Interestingly, the frequency of AVSD (10.5%) in our study was higher than in any other African study. In general, the frequency of AVSD in studies from developing countries is reported to be between 3.5 and 8.8% [17, 21, 22]. The high prevalence of AVSD, mainly in association with DS, was a constant finding throughout the consecutive years. The prevalence of DS in Tanzania is not known, but studies from Nigeria and South Africa have stated that the prevalence of DS in Sub Saharan Africa is exceeding the reported prevalence of 1 in 750 live births in western countries [23, 24]. This may be influenced by prenatal diagnosis and termination of pregnancy in the western countries [25]. Furthermore, a recent study from Nigeria examined the prevalence of CHD in children with DS and found a prevalence of 75%, which is significantly higher than reported by others [26].

Overall, the rate of associated extracardiac malformations corresponds well with the findings of other studies [25, 27, 28].

RHD presented as the leading cause of AHD in children in our study similar to what is consistently reported worldwide [13]. It is furthermore very likely that the condition is widely underreported in our review as the vast majority of patients was diagnosed with advanced findings and we didn’t detect any subclinical RHD as reported in other reviews [11].

Echocardiography is a valuable diagnostic tool as it is non-invasive and cost-effective. The widespread use of it should be enhanced with more paediatricians being trained in this technique, especially in settings with low resources. In a first step paediatricians trained and supervised by paediatric cardiologists to perform echocardiography in the rural areas with mobile echocardiography equipment has to be the goal to diagnose CHDs and AHDs in a large number and in a reasonable time.

Limitations of the study: This study is a single centre, hospital based review and not a community based study. Therefore, the study does not provide information on the prevalence of heart diseases in the general paediatric population of Tanzania. Furthermore, it is a retrospective review of the echocardiography registry, which contains some incomplete data or missing information. Diagnostic tools like cardiac catheterization or cardiac CT scan were not available in Tanzania during the time of the study period so that cardiac diagnoses were mainly based on echocardiographic findings. Long-term outcome of the patients is incomplete as a substantial number of patients were lost to follow-up.

## Conclusion

The spectrum of congenital and acquired heart diseases presenting to the paediatric cardiac echocardiography clinic of BMC is corresponding well to the findings reported in other countries from the African continent.

The majority of children with heart disease presenting to our clinic is in need of surgical or interventional treatment, which is not readily available. Furthermore, diagnosis is often delayed and as a consequence a significant number of patients show severe complications and an advanced stage of the heart disease on initial diagnosis. Untreated heart diseases contribute substantially to morbidity and mortality during infancy and childhood. Adequate local cardiac services should be established and strengthened.

Echocardiography is a valuable diagnostic tool as it is non-invasive and cost-effective. The widespread use of it should be enhanced with more paediatricians being trained in this technique, especially in settings with low resources.

Children diagnosed with CHD should be carefully examined for extracardiac malformations due to the significant association between CHD and extracardiac malformations.

All children with DS should routinely receive a cardiac examination because of the high association of CHD. A further study would be needed to determine the prevalence of CHD in children with DS in Tanzania.

RHDs are certainly underreported as most patients were diagnosed with advanced findings and no cases of subclinical RHD were detected. Further population based prevalence studies are required to determine the real burden of this disease and to guide preventive programmes.

## Data Availability

The datasets analysed during the current study are available from the corresponding author upon reasonable request.

## Abbreviations

AHD: Acquired heart disease
AVSD: Atrioventricular septal defect
BMC: Bugando medical Centre
CHD: Congenital heart disease
DORV: Double outlet right ventricle
DS: Down syndrome
PDA: Patent ductus arteriosus
RHD: Rheumatic heart disease
TAPVR: Total anomalous pulmonary venous return
ToF: Tetralogy of Fallot
VSD: Ventricluar septal defect
